# Treatment of Rapamycin and Evaluation of an Autophagic Response in the Gut of *Bactericera cockerelli* (Sulč)

**DOI:** 10.3390/insects14020142

**Published:** 2023-01-31

**Authors:** Junepyo Oh, Cecilia Tamborindeguy

**Affiliations:** Department of Entomology, Texas A&M University, College Station, TX 77843, USA

**Keywords:** potato psyllid, ‘*Candidatus* Liberibacter solanacearum’, zebra chip, autophagy, programmed cell death

## Abstract

**Simple Summary:**

In North America, the Gram-negative plant bacterial pathogen ‘*Candidatus* Liberibacter solanacearum’ (Lso) imposes a serious threat to most solanaceous crops, and in potato, it causes zebra chip disease. Lso is transmitted by the potato psyllid, *Bactericera cockerelli* (Šulc), in a circulative and persistent manner. Our previous study showed that autophagy might be involved in the transmission, but tools to evaluate autophagy in this species have not been validated. The results showed that a 24 h ingestion of rapamycin resulted in increased mortality and could induce autophagy as determined via microscopy and measurement of the autophagic flux by Western blot. This study validated rapamycin as an autophagy inducer and validated tools to assess the response in the gut of adult potato psyllid.

**Abstract:**

Autophagy is a catabolic process that results in the autophagosomic–lysosomal degradation of bulk cytoplasmic content, abnormal protein aggregates, and excess of/or damaged organelles to promote cell survival. Autophagy is also a component of innate immunity in insects and is involved in the clearance of pathogens, including bacteria. The potato psyllid, *Bactericera cockerelli*, transmits the plant bacterial pathogen ‘*Candidatus* Liberibacter solanacearum’ (Lso) in the Americas and causes serious damage to solanaceous crops. Our previous studies showed that autophagy could be involved in the psyllid response to Lso and could affect pathogen acquisition. However, the tools to evaluate this response have not been validated in psyllids. To this end, the effect of rapamycin, a commonly used autophagy inducer, on potato psyllid survival and the expression of autophagy-related genes was evaluated. Further, the autophagic activity was assessed via microscopy and by measuring the autophagic flux. Artificial diet-feeding assays using rapamycin resulted in significant psyllid mortality, an increase in the autophagic flux, as well as an increase in the amount of autolysosomes. This study represents a stepping stone in determining the role of autophagy in psyllid immunity.

## 1. Introduction

Rapamycin, also known as sirolimus, is one of the immunosuppressive drugs that inhibit the mechanistic target of rapamycin complex one (mTORC1), which affects the regulation of many fundamental cell processes, such as cell growth and metabolism [1]. Rapamycin has been used to induce autophagy in a diverse range of organisms, from yeast to mammals [2].

Autophagy, a conserved cellular degradation process within lysosomes for unnecessary and/or damaged cellular components, has been suggested to protect cells from nutritional or cellular stress [3,4]. In insects, autophagy is involved in many biological processes, including development, aging, and neurodegeneration [5]. In general, autophagy is characterized by the formation of autophagosomes, which are double-membrane vesicles that isolate the cellular components, and subsequently, autolysosomes are formed by the fusion of lysosomes for degradation [6]. Autophagy also plays an important role in innate immune responses to viral, bacterial, and fungal pathogens in many invertebrates [7]. For example, in Drosophila, autophagy is crucial for defense against various pathogens, such as the Gram-positive bacterium *Listeria monocytogenes* [8] and the negative-sense rhabdovirus vesicular stomatitis virus [9]. Interestingly, an increasing number of studies have found that vector-borne plant viruses can induce autophagy in hemipteran insects. In these cases, the introduction of autophagy may serve as a mechanism to defend the insect from pathogen invasion, or it may be the result of the manipulation of the vector defenses by pathogens for their own benefit. For example, infection with the tomato yellow leaf curl virus (TYLCV) activates autophagy in the Middle East Asia Minor 1 (MEAM1) species of *Bemisia tabaci*, resulting in lower transmission, whereas inhibiting this response increases viral transmission [10,11]. On the other hand, autophagy induction in the leafhopper vector *Recilia dorsalis* increases the spread and/or transmission of the rice gall dwarf virus (RGDV) and rice dwarf virus (RDV) [12]. However, fewer studies have investigated the activation or inhibition of autophagy in response to vector-borne bacterial plant pathogens in hemipterans and their effects on bacterial acquisition and transmission.

The potato psyllid, *Bactericera cockerelli* (Sulč), is a polyphagous, phloem-feeding hemipteran insect that feeds on a wide variety of host plant species, such as tomatoes, potatoes, and other solanaceous crops. While this insect is native to the southern USA and northern Mexico, today, it is found in the western half of the USA, parts of Canada, Mexico, Central America, New Zealand, parts of Australia, and more recently, it has been found in South America [13,14,15,16,17]. The symptoms caused by potato psyllid feeding in potatoes include yellowing and chlorosis of the foliage, and a reduction in potato tuber size [18,19,20]. The potato psyllid transmits ‘*Candidatus* Liberibacter solanacearum’ (Lso) [21]. In the Americas, Lso is the causative agent of zebra chip, a serious disease in potatoes, and it also causes severe damage to other solanaceous crops [16]. Lso is transmitted in a persistent and circulative manner. The psyllid gut is the first organ that Lso invades, and it can act as a barrier to Lso transmission [22]. Our previous research demonstrated that potato psyllid has 19 autophagy-related genes (ATGs), and ATGs were upregulated in the potato psyllid gut after Lso infection [23]. However, tools to evaluate and manipulate the autophagic response have not been validated in potato psyllids. These tools are needed to determine if autophagy is indeed induced in response to Lso and whether this response is to the advantage of the pathogen or the vector. In the present study, we evaluated the effect of rapamycin-containing artificial diets, a commonly used autophagy inducer, on potato psyllid survival and the expression of ATGs. Further, we assessed the autophagic activity via microscopy, and we evaluated the autophagic flux, which measures the autophagic degradation activity. This study is the first step in determining the role of autophagy in psyllid immunity.

## 2. Materials and Methods

### 2.1. Plant and Insects

Tomato (*Solanum lycopersicum* L. ‘Moneymaker’; Thompson and Morgan Inc., Jackson, NJ, USA) seeds were sown in pots containing Sun Gro Sunshine LP5 mix (Bellevue, WA, USA). The obtained plants were fertilized twice a week with Miracle-Gro Water-Soluble Tomato Plant Food at the label rate (18-18-21 NPK; Scotts Miracle-Gro Company, Marysville, OH, USA).

A potato psyllid colony was maintained on tomato plants in insect cages (24 × 13.5 × 13.5 cm, BioQuip, Compton, CA, USA) at room temperature 24 ± 1 °C and a photoperiod of 16:8 h (L:D). 

### 2.2. Feeding Bioassay and Induction of Autophagy

**Figure 1:**
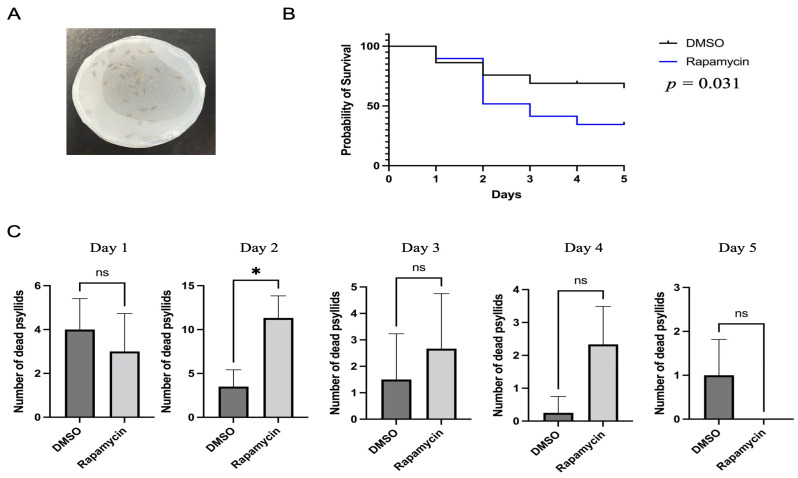
Analysis of potato psyllid mortality caused by feeding on rapamycin-containing diets. (**A**). Apparatus for the feeding assays. The liquid diet was placed between two Parafilm sheets that covered the chambers. (**B**). Mortality of potato psyllids following 24 h feeding on artificial diet without (control) or with 10 µM rapamycin. The reported *p*-value refers to the log-rank test. (**C**). Analysis of the daily mortality of psyllids. All values are represented as mean ± SD (* *p* < 0.05).

For the psyllid feeding bioassays, 15% (w:v) sucrose and 1x phosphate-buffered saline (1x PBS) solution (Sigma-Aldrich, St. Louis, MO, USA) were used as a liquid diet. Rapamycin at a concentration of 10 μM was added into the diet by dissolving it in dimethyl sulfoxide (DMSO) [11,24]. The experiment also included control diets with an equivalent amount of DMSO. Pools of psyllid adults were collected from the potato psyllid colony and placed in plastic feeding chambers (h = 2 cm, Φ = 3 cm) covered by two sheets of Parafilm with 100 μL of the liquid diet between the two layers (Figure 1A). The insects were allowed to feed from the liquid diet for 24 h, then they were transferred to healthy tomato plants. Psyllid survival was monitored every 24 h for five days. Three replicates, consisting of 30 psyllid individuals from the potato psyllid colony each, were analyzed.

### 2.3. Gene Expression of Autophagy-Related Genes

Data mining of the potato psyllid transcriptome identified 19 ATGs [23,25]. For the gene expression experiment, pools of psyllid adults (~50 insects) were allowed to feed on diets containing 10 μM of rapamycin or control diets (DMSO only, without rapamycin) for 24 h. Then, the psyllid guts from live insects were dissected in 1×PBS under the Leica EZ4W0037 stereomicroscope (Leica Microsystems, Germany). Dissections were performed following the dissecting methods in [26]. RNA from pools of 30 psyllid guts were purified using the Bead Mill Tissue RNA Purification kit (Omni International, Kennesaw, GA). The cDNA was synthesized using a Verso cDNA Synthesis Kit (Thermo, Waltham, MA, USA) and anchored oligo (dT) primers as described in the kit’s manual. Genomic DNA was removed by the DNase I treatment with Turbo DNase (Ambion, Austin, TX, USA). For each experiment, three replicates were analyzed, and each replicate had 30 psyllid individuals. 

The expression of nine autophagy-related genes was evaluated by quantitative PCR (qPCR). The ATGs tested were ATG1, ATG3, ATG4B, ATG5, ATG7, ATG8, ATG10, ATG12, and ATG16. ATG1 is a serine/threonine-protein kinase ULK2-like isoform 1, which contributes to autophagy initiation and phagophore formation. ATG3 is an E2-like enzyme for the ATG8 lipidation process. Cysteine protease ATG4B cleaves the precursor ATG8 protein at the C-terminal, thereby initiating autophagosome formation. ATG5 is an autophagy protein that is essential for phagophoric membrane extension; it is activated by ATG7 and forms a complex with ATG12 and ATG16 (ATG12-ATG5/ATG16). ATG8 is a ubiquitin-like protein required for the formation of autophagosomal membranes after transient conjugation. The ATG12-ATG5/ATG16 complex is one of the important conjugation systems for ATG8-I conjugation to phosphatidylethanolamine to form ATG8-II. ATG10 functions as an E2-like enzyme, catalyzing the conjugation of ATG12 to ATG5. All qPCR reactions were performed using a PowerUp SYBR Green Master Mix (Applied Biosystems, Waltham, MA, USA) following the manufacturer’s instructions. Each reaction included 10 ng of cDNA, 250 nM of each primer [22], and 5 μL of PowerUp SYBR Green Master Mix (Applied Biosystems); nuclease-free water was used to adjust the volume to 10 μL. The qPCR program was 95 °C for 2 min followed by 40 cycles of 95 °C for 5 sec and 60 °C for 30 sec. Three technical replicates were performed for each reaction, and negative controls (no cDNA) were included in each run. The qPCR reactions were run in a QuantStudio 6 Flex Real-Time PCR System (Thermo Fisher Scientific, Waltham, MA, USA). The relative expression of the candidate genes was calculated using the delta delta Ct method [27] with ribosomal protein subunit 18 (GenBank KT279693) [28] as the reference gene.

### 2.4. Lysotracker Green DND-26 Staining

To visualize the induction of autophagy in the potato psyllid gut following rapamycin ingestion, the gut of adult psyllids that had fed on control or rapamycin-containing diets for 24 h were dissected as previously described and transferred to 50 µL dye solution containing 1 µM of Lysotracker Green DND-26 (Invitrogen, Carlsbad, CA, USA) and 10 µg/mL of DAPI (Sigma-Aldrich). The guts were incubated in the dye solution as described in [29]. The guts were mounted using 1×PBS and documented immediately under a fluorescent microscope (Axio Imager A1 microscope, Carl Zeiss Microscopy, White Plains, NY, USA). Three replicates were analyzed, each containing at least fifteen guts. The signals obtained from Lysotracker were compared in multiple 50 µm^2^ image fields from control and rapamycin-treated guts using the ImageJ software. A minimum of 6 images were used for measurement for the analysis.

### 2.5. Protein Extraction and Western blotting

The autophagic flux is characterized by ATG8 lipidation, which is the conversion of ATG8-I to ATG8-II. These proteins are necessary for the elongation and maturation of autophagosomes, and the conversion ratio reflects the amount of autophagosomes that subsequently fuse with lysosomes for degradation. To measure the autophagic flux, the total proteins from 20 psyllid guts that fed on control or rapamycin-containing diets for 24 h were purified using the RIPA buffer (Invitrogen, Carlsbad, CA, USA) supplemented with protease inhibitor tablets (Roche Diagnostics, Basel, Switzerland). The total protein extracts were quantified by the Bradford assay. The proteins were separated on a 4–12% Bis-Tris NuPage gel (Invitrogen) by loading approximately 10 μg of the proteins mixed with 4X SDS sample buffer onto each lane. Then, following standard blotting procedures, the proteins were transferred to an Immobilon-P PVDF membrane (Millipore-Sigma, Burlington, MA, USA) and visualized using a Pierce™ Reversible Protein Stain Kit for PVDF Membranes (Thermo Fisher Scientific). The membrane was blocked with 5% dry milk in the TBST buffer at room temperature for 1 h, followed by incubation with the primary antibody anti-GABARAP+GABARAPL1+GABARAPL2 (ATG8) (ab109364; Abcam, Cambridge, United Kingdom) or anti-Actin (MA1-744; Thermo Fisher Scientific) at 4 °C overnight. After washing off the primary antibody, the blot was incubated with the anti-rabbit IgG secondary antibody (Sigma-Aldrich) or HRP conjugated goat anti-mouse IgG (H+L) secondary antibody (Invitrogen) at room temperature for 1 h. The SuperSignal West Pico substrate (Invitrogen) was used to detect the signal on an iBright 1500 imaging system (Thermo Fisher Scientific). ImageJ software was used to analyze and quantify the signal intensity to measure the autophagic flux by calculating the ratio of the ATG8-II/ATG8-I. The experiment was carried out twice in triplicate.

### 2.6. Statistical Analyses

JMP Version 16 (SAS Institute Inc., Cary, NC, USA) and GraphPad Prism 9 Software (GraphPad Software, San Diego, CA, USA) were used for all data analyses. For psyllid survival analyses, Kaplan–Meier survival curves and log-rank tests were used. Furthermore, the mortality of potato psyllids fed on rapamycin-containing or control diets each day was compared using the Mann–Whitney U-test. The effects of rapamycin on the expression of ATGs, the lysosomal activity, and the autophagic flux were determined with Student’s t-tests. All values are represented as mean ± SD (* *p* < 0.05, ** *p* < 0.01, *** *p* < 0.001, and **** *p* < 0.0001).

## 3. Results

### 3.1. Rapamycin Significantly Increased Mortality

The mortality of potato psyllids was monitored following feeding on control or rapamycin-containing diets. The results showed that rapamycin significantly increased psyllid mortality when compared to the control diets lacking rapamycin (Figure 1B, log-rank test: *p* < 0.05). After two days of feeding on the rapamycin-containing diets, the number of dead potato psyllids was substantially greater than those on control diets (Figure 1C). By day 5, on average, over 65% of psyllids were still alive in the control treatment, while 34% of potato psyllids were alive in the rapamycin-containing treatment (Figure 1B). 

### 3.2. Expression of Autophagy-Related Genes

**Figure 2:**
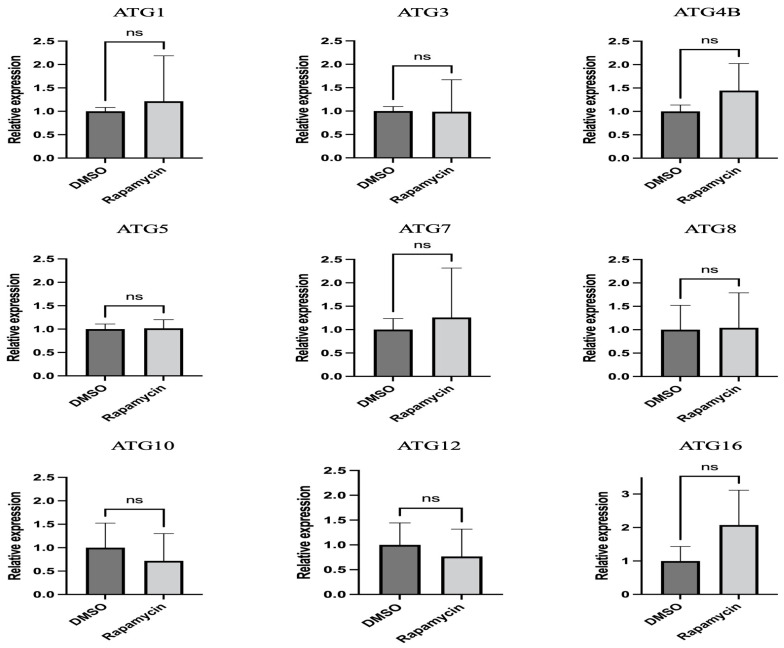
Relative expression of autophagy-related genes in midguts of potato psyllids after exposure to control and rapamycin-containing diets for 24 h. All values are represented as mean ± SD.

Gene expression results showed that none of the tested ATGs were significantly regulated after 24 h (Figure 2). 

### 3.3. Rapamycin Feeding Increased Lysosomal Activity

**Figure 3:**
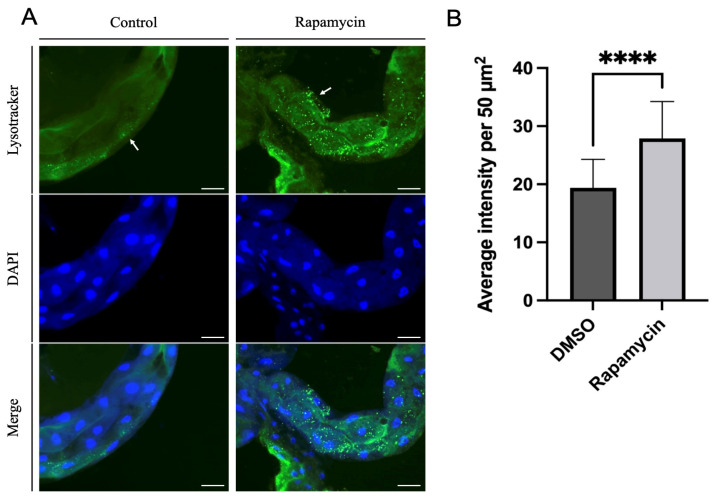
(**A**). Staining of lysosomes and autolysosomes (green) and nuclei (blue) in psyllid guts. More Lysotracker signals indicating increased lysosomal activity were observed in the midguts of psyllids fed on rapamycin-containing diets for 24 h compared to the control midguts. Left panel: control diets; right panel: rapamycin-containing diets. (**B**). Average intensity per 50 µm^2^ of Lysotracker in control and rapamycin-treated midguts (n = 18 fields). Arrow indicates lysosomal signals. All values are represented as mean ± SD (**** *p* < 0.0001). Scale bar = 50 µm.

Potato psyllids were allowed to feed on control or rapamycin-containing diets for 24 h, then their guts were dissected and stained with Lysotracker prior to imaging. An increase in Lysotracker-positive lysosomes and autolysosomes was observed in the midgut of psyllids fed on the rapamycin-containing solution, indicating autophagy induction (Figure 3A shows representative images of the psyllid guts from both treatments). The quantification of the average intensity per 50 µm^2^ in midguts of potato psyllids showed significantly higher intensity in potato psyllids that fed on rapamycin (Figure 3B). 

### 3.4. Rapamycin Feeding Increased the Autophagic Flux

**Figure 4:**
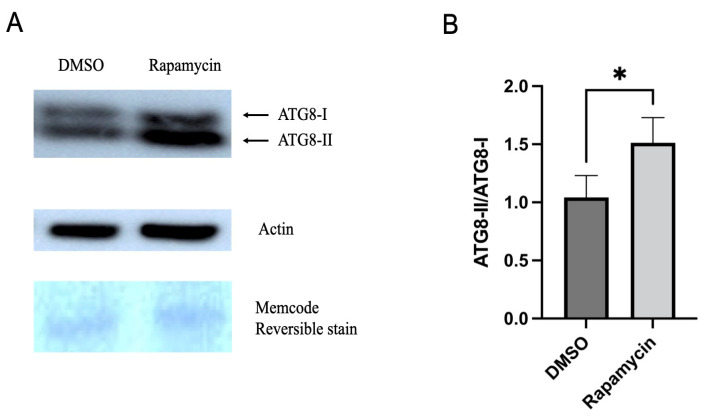
(**A**). Western blot analysis with anti-ATG8 antibody from protein extracts of 20 guts; bands corresponding to ATG8-I and ATG8-II were detected. (**B**). Ratio of ATG8-II/ATG8-I showing the conversion rate of ATG8-I to ATG8-II shown in A. ATG8-II represents lipidated ATG8-I. All values are represented as mean ± SD (* *p* < 0.05).

The result of the Western blot showed an accumulation of ATG8-II in the guts of psyllids fed on rapamycin-containing diets for 24 h compared to the control group (Figure 4A). The accumulation of ATG8-II was compared with ATG8-I and showed a significantly increased accumulation in the guts of psyllids that were fed on the rapamycin-containing diet, indicating the autophagic flux (Figure 4B). Additional western image is available in Figure S1.

## 4. Discussion

Rapamycin is an autophagy inducer that activates autophagy by inhibiting the mechanistic target of rapamycin complex 1 (mTORC1) [30]. Rapamycin can form a high-affinity complex with the intracellular protein FKBP12; the peptidyl-prolyl cis-trans isomerase and the FKBP12/rapamycin complex can prevent the association of raptor with mTORC1, which is essential for mTORC1 activity and regulation in eukaryotes [31,32,33]. Autophagy is an intracellular self-degradation process and is highly conserved in eukaryotes, removing and recycling unwanted cell components to relieve stress in a lysosome-dependent manner [4,34]. Autophagy also plays an important role in innate immunity. Many studies have demonstrated the role of autophagy in combating intracellular microbes in mammals [35], and its role in immunity has also been identified in invertebrate model organisms, such as *Drosophila melanogaster* and *Caenorhabditis elegans* [7]. However, autophagy could also be manipulated by microbes for their own benefits, for example, by promoting infection and transmission by hemipteran vectors [12].

In our previous study, we identified 19 ATGs and evaluated the expression pattern of those ATGs in the gut of potato psyllids upon Lso acquisition and found many of those genes to be regulated [23]. Therefore, we hypothesized that autophagy could be used in psyllids to stop infection, but Lso could also manipulate it to promote infection. To test this hypothesis, we first needed to validate with psyllids the tools for autophagy evaluation. In the present study, we found that rapamycin was highly toxic to potato psyllids and caused significant mortality. Furthermore, we found that rapamycin caused higher mortality in psyllids after two days of feeding compared to control psyllids (Figure 1). This is the first artificial diet study using rapamycin in potato psyllids, which showed that 10 µM rapamycin, a dose previously used to induce autophagy in whiteflies, another phloem-feeding hemipteran [11,36], is toxic to potato psyllids. However, when carrot psyllids were allowed to feed on leaves placed in a microcentrifuge tube containing 10 μM rapamycin, low mortality was recorded [24]. 

ATGs are necessary for the formation of autophagosomes. The number of ATGs varies in eukaryotes because ATGs have been duplicated and lost during evolution. For example, several ATG8 genes can be found in mammals, whereas there is only a single ATG8 gene in yeast and fungal species [37,38]. Several lines of study have demonstrated the importance of ATGs in immunity against various pathogens by RNAi silencing. For instance, silencing ATG3 or ATG9 in the planthopper *Sogatella furcifera* increased the propagation and transmission rates of the southern rice black-streaked dwarf virus (SRBSDV) [39]. In *Tenebrio molitor*, the depletion of ATG3 and ATG5 by RNAi decreased the survival ability of *T. molitor* larvae against *L. monocytogenes* [40]. Another study on carrot psyllid, *Bactericera trigonica*, which can transmit Lso haplotype D, showed that the induction of autophagy using rapamycin significantly decreased Lso titer and upregulated ATG2, ATG5, and ATG16 in the gut. In contrast, the inhibition of autophagy using thapsigargin significantly increased the Lso haplotype D titer and reduced the expression of ATG2, ATG5, and ATG16 [24]. Although changes in the expression of ATGs cannot be used to confirm autophagy regulation, we expected the regulation of tested ATGs in the gut at the transcriptional level after feeding on rapamycin. However, the results showed that none of the tested genes were significantly regulated after 24 h of feeding on diets containing 10 μM rapamycin (Figure 2). This discrepancy might be because the transcriptional regulation of each gene is different during autophagy. Psyllid mortality was greater when allowed to feed on diets containing rapamycin for 36 h rather than 24 h (data not shown); thus, it is possible that insects used for this assay had ingested lower amounts of rapamycin. None of the genes were regulated after 24 h feeding on rapamycin. At that point in time, there was no mortality of psyllids, and it is possible that rapamycin ingestion had been low. The presence of autolysosomes was markedly increased in the midguts of rapamycin-fed psyllids (Figure 3). This is consistent with the study previously discussed which showed increased lysosomal activity after *B. trigonica* were fed on leaves submerged in rapamycin-containing media [24]. Therefore, measuring increased lysosomal activity could be used to test the autophagic response in potato psyllids. Monitoring autophagy has been extensively discussed in cultured cells and model organisms [41]. ATG8 lipidation, the conjugation of PE to ATG8-I to form ATG8-II, is a hallmark of autophagy where these proteins are required for the formation of autophagosome because the amount of ATG8-II reflects the number of autophagosomes and autophagy-related structures [42]. However, no study has measured the autophagic flux in potato psyllids. Here, first, we validated that the antibody ab109364 (Abcam) can detect psyllid ATG8-I and ATG8-II, and second, demonstrated an increased accumulation of ATG8-II in the gut of psyllids after feeding on rapamycin-containing diets (Figure 4).

## 5. Conclusions

In summary, we demonstrated that the ingestion of 10 μM rapamycin caused high mortality in potato psyllids. While this treatment was effective to validate tools to analyze autophagy in this species, it cannot be used to trigger autophagy and study Lso transmission in this species, which requires several days for circulation to healthy psyllids capable of feeding on plants and inoculating the pathogen. In addition, tools for evaluating autophagy in psyllids were validated. The gene expression of ATGs was not enough to test the autophagic response, but combining Lysotracker Green-DND 26 to evaluate the lysosomal activity and Western blotting to measure the conversion from ATG8-I to ATG8-II could be used to evaluate the autophagic response in psyllids. Overall, this study is the first step toward determining the role of autophagy in psyllid immunity against the intracellular bacterium Lso.

## Data Availability

The data presented in this study are available in this article.

## Supplementary Materials

The following supporting information can be downloaded at: https://www.mdpi.com/article/10.3390/insects14020142/s1, Figure S1: Western blot detection of ATG8-I and ATG8-II proteins in psyllid guts with anti-ATG8 antibody and densitometry readings for ATG8-I and ATG8-II.
