# Supplementary material for: Treatment of Rapamycin and Evaluation of an Autophagic Response in the Gut of Bactericera cockerelli (Sulč)
Source: Insects. 2023 Jan 31;14(2):142. doi: 10.3390/insects14020142 (PMC9958837; doi:10.3390/insects14020142)
Supplement: Supplementary file 1 [file insects-14-00142-s001.zip › insects-2107116-supplementary.pdf]

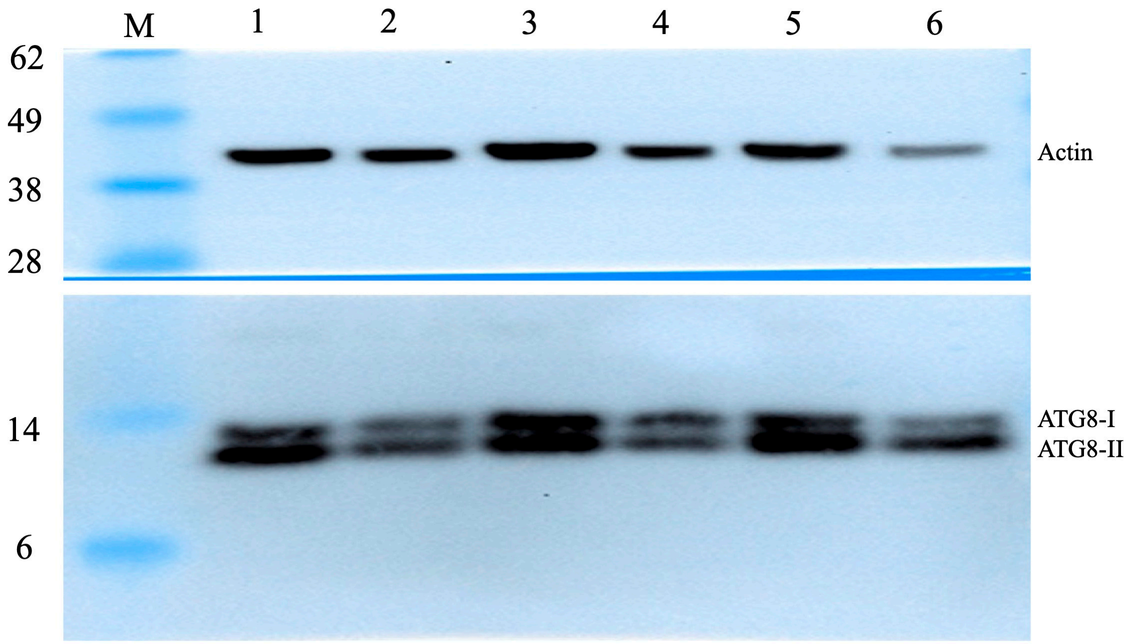

	1	2	3	4	5	6
ATG8-I	81131.597	67729.002	109281.869	79089.491	85835.467	68592.943
ATG8-II	132436.317	79935.6271	137862.774	65291.4538	140949.753	76815.4212
Ratio (ATG8-II/ATG8-I)	1.632364232	1.180227447	1.261533825	0.82553893	1.642092225	1.11987353

Figure S1. Western blot detection of ATG8-I and ATG8-II proteins in psyllid guts with anti-ATG8 antibody and densitometry readings for ATG8-I and ATG8-II. Potato psyllids were fed on rapamycin-containing diets or DMSO-containing diets for 24 h. Lane M: molecular weight marker (SeeBlueT Plus2 Pre-stained Protein Standard, Invitrogen); Lane 1: Rapamycin-treated guts (replicate 1); Lane 2: DMSO-treated guts (replicate 1); Lane 3: Rapamycin-treated guts (replicate 2); Lane 4: DMSO-treated guts (replicate 2); Lane 5: Rapamycin-treated guts (replicate 3); Lane 6: DMSO-treated guts (replicate 3).
